# Photon-counting CT of elbow joint fractures: image quality in a simulated post-trauma setting with off-center positioning

**DOI:** 10.1186/s41747-023-00329-w

**Published:** 2023-03-27

**Authors:** Lena Sonnow, Nigar Salimova, Lea Behrendt, Frank K. Wacker, Marcus Örgel, Jochen Plagge, Friederike Weidemann

**Affiliations:** 1 Department of Diagnostic and Interventional Radiology, Hannover Medical School, Hannover, Germany; 2 Department of Trauma Surgery, Hannover Medical School, Hannover, Germany; 3 Department of Orthopedic Surgery, Hannover Medical School at Diakovere Annastift, Hannover, Germany

**Keywords:** Cadaver, Elbow, Olecranon process, Musculoskeletal system, Tomography (x-ray computed)

## Abstract

**Background:**

Photon-counting detector computed tomography (PCD-CT) has the potential to provide superior image quality compared to energy-integrating detector computed tomography (EID-CT). We compared the two systems for elbow imaging in off-center arm positioning, 90° flexion, and cast fixation in a simulated post-trauma setting.

**Methods:**

The institutional review board approved the study protocol. In a cadaver study, an olecranon fracture was artificially created in ten whole arm specimens. Two different scanning positions were evaluated: (a) arm overhead; and (b) arm on top of the abdomen of a whole-body phantom. The ultra-high resolution mode with three dose protocols and two reconstruction kernels was applied. Two blinded radiologists independently evaluated fracture and trabecular bone delineation. Signal-to-noise ratio (SNR), contrast-to-noise ratio (CNR), and cortical sharpness measurements were performed. Cohen κ correlations, Mann-Whitney *U* and Wilcoxon signed rank tests were used. A *p* value lower than 0.05 was considered statistically significant.

**Results:**

Dose-equivalent PCD-CT scans were rated better for fracture and trabecular bone evaluation (*p* < 0.001). SNR, CNR, and cortical sharpness were higher for all diagnostic (Br76) PCD-CT images (*p* < 0.001). The arm position had less effect on image quality in the PCD-CT compared to the EID-CT. The use of a sharp bone kernel (Br89) improved image quality ratings for PCD-CT. In the low-dose scan mode, PCD-CT resulted in more diagnostic scans (75%) compared to EID-CT (19%).

**Conclusions:**

PCD-CT provided superior objective and subjective image quality for fracture and trabecular bone structures delineation of the elbow compared to EID-CT in a typical post-trauma setting.

**Key points:**

• Photon-counting detector computed tomography (PCD-CT) preserved high image quality in elbow imaging with off-center positions.

• PCD-CT was advantageous for bone evaluation in trauma elbows.

• PCD-CT ultra-high-resolution mode and very sharp reconstruction kernels facilitated higher image quality.

## Background

Computed tomography (CT) of elbow fractures is usually performed for detailed evaluation beyond radiography and accurate fracture classification. CT reveals all fracture fragments in detail and exposes the complexity and severity of the injury [1]. In evaluating olecranon fractures, emphasis should be placed on describing the degree of displacement and the presence of comminution, which are key determinants for the treatment approach. The CT scan requires high quality images with multiplanar reconstructions and the possibility of three-dimensional (3D) evaluation of anatomy and fracture morphology.

A major challenge for CT scans of the elbow joint remains an appropriate patient positioning for optimal image quality and reduced radiation exposure. Generally recommended is an elevated arm, in either prone or supine overhead positions. A reduced range of motion of the shoulder joint can require an arm positioning on top of the patient’s abdomen in supine position. The major disadvantage of this position is a radiation dose increase and low image quality, especially in obese patients. An additional restriction in a post-trauma setting is the cast-fixation of the elbow joint in 90° flexion, possibly increasing imaging artifacts. Recent practical recommendations for ultra-high resolution (UHR) CT of joints emphasize patient centering, which is an important factor affecting spatial resolution [2]. Moreover, a mis-centered patient can lead to an undesired increase in both, radiation dose and image noise [3].

In current energy-integrating detector (EID)-CT scanners, a dedicated UHR imaging mode implies the insertion of a movable grid and comb filter in front of the multi-row detector. Both techniques are associated with an increase in radiation dose to achieve comparable noise characteristics to a normal scan mode [4]. Other important technical aspects include bowtie filter geometry as well as angular sampling principles, which are optimized for evaluating round or elliptical objects at the center of the gantry [5]. Hence, image quality can be considerably reduced in the periphery at the same radiation dose.

Photon-counting detector (PCD)-CT have recently become available for clinical applications. PCDs are composed of semiconductors converting x-rays into an electric signal. The number of incoming photons is counted and photon energy can be measured without a photon being converted into light [6]. Recent studies demonstrated superior capabilities and diagnostic performance of PCD-CT compared to conventional EID-CT [7–10]. In musculoskeletal imaging, the UHR capability and the electronic noise reduction techniques improve bone and fracture visualization. Recent technical assessments and initial patient experience demonstrated an in-plane spatial resolution of 125 μm [11, 12] and lower image noise [13], offering a better assessment of bone microstructure at similar dose levels. However, due to the innovativeness of the technology there is limited information on clinical benefits of PCD-CT for joint imaging. We assume, that the PCD-CT offers the advantage of better image quality for bone and fracture assessment especially in challenging post-traumatic situations with off-centered patient positions and undesired overlay effects with possible, inhomogeneous radiation dose decrease.

The objective of this study is to compare the imaging performance of a PCD- and an EID-CT system for diagnostic evaluation of elbow joint fractures with off-center arm positioning, 90° flexion and cast fixation in a simulated post-trauma setting.

## Methods

This study was carried out following the ethical standards laid down in the 1964 Declaration of Helsinki and its later amendments. The institutional review board approved the study protocol (approval number: 10117_BO_K_2021). Body donors consented during their lifetime to the use of their bodies for study and research purposes.

### Cadaver preparation and positioning

Ten cadaveric whole arm specimens exarticulated at the glenohumeral joint were obtained from the Department of Anatomy of our university.

To simulate an elbow fracture, we performed a chevron osteotomy of the olecranon: a standard posterior incision of the olecranon was made with a slightly curved cut at the tip of the olecranon. The incision started at the distal third of the humeral shaft and ended at the proximal third of the ulnar bone. Medial and lateral full-thickness fasciocutaneous flaps were prepared. To expose the olecranon, the anconeus muscle was separated laterally and the origin of the flexor carpi ulnaris muscle medially.

The osteotomy is recommended at the bare area of the olecranon to avoid cartilage damage. Therefore, approximately 2 cm distally of the tip of the olecranon a 2-mm bicortical hole was drilled to mark the apex of the chevron osteotomy. To simulate a maximal fracture-like osteotomy, a 6 mm osteotome was used to perform the osteotomy up to the subchondral bone. The subchondral bone was fractured by external force by the surgeon due to maximal flexion of the elbow. The integrity of the osteotomy was verified using fluoroscopy. For wound closure Prolene 2-0 was used in uninterrupted suture technique.

**Fig. 1:**
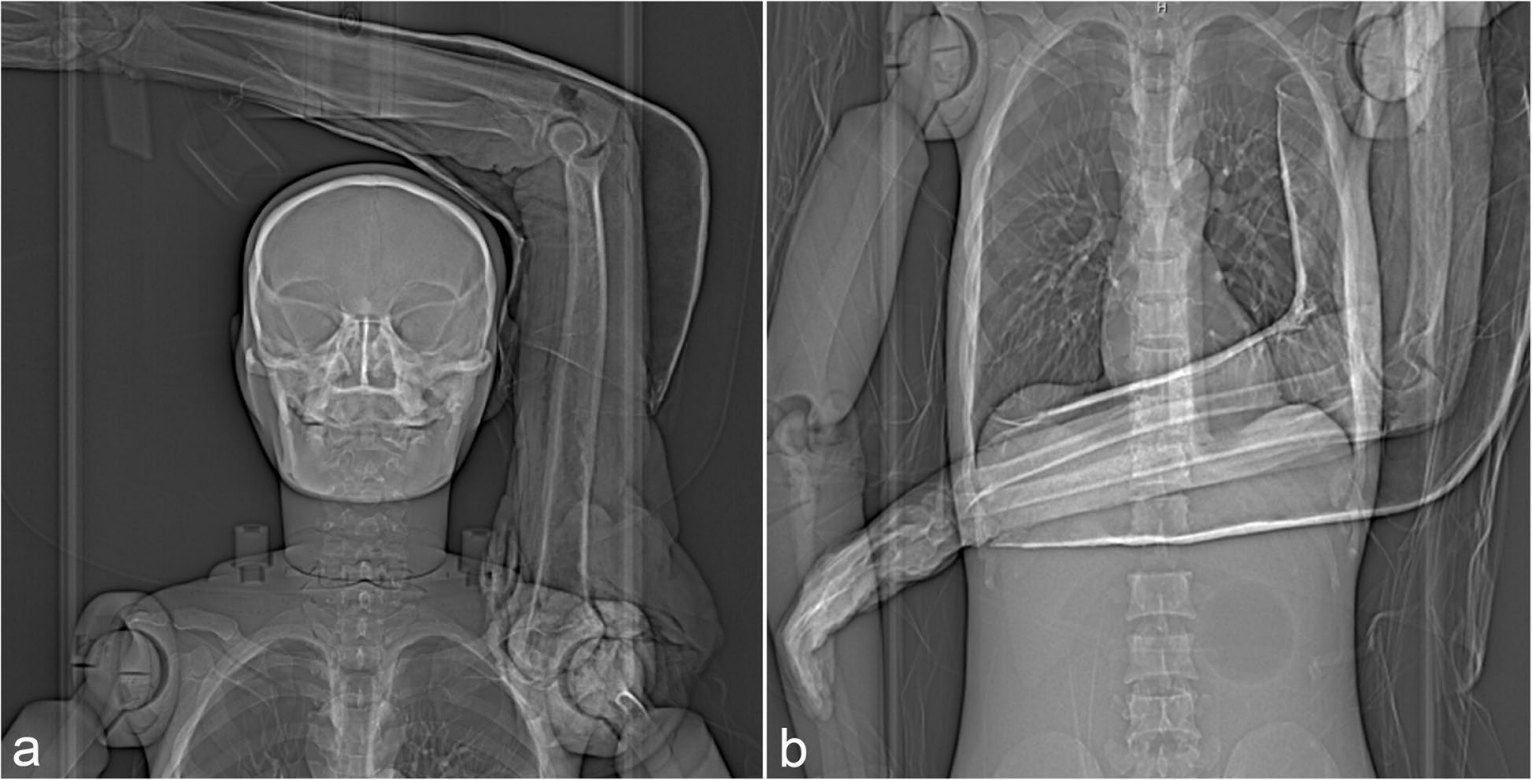
Simulation of post-trauma scanning conditions with positioning of the cadaveric arm either above the head (**a**) or on the abdomen (**b**) of a whole-body phantom. A cast fixates the elbow joint in 90° flexion. CT-scouts for orientation

A whole-body phantom (PBU-60, Kyoto Kagaku, Fushimi-ku, Kyoto, Japan) was used in supine position. Two different scanning positions were simulated with the cadaveric arms either overhead or on top of the abdomen in 90° flexion of the elbow joint and with an attached cast (Fig. 1).

### Scanner parameters and image acquisition

All specimens were examined with a clinical PCD-CT system (NAEOTOM Alpha, version syngo CT VA40A, Siemens Healtheeners GmbH, Forchheim, Germany) and a dual source EID-CT scanner (SOMATOM Force, Siemens Healtheeners GmbH, Forchheim, Germany). For both scanners we chose three different dose-protocols with a fixed reference tube voltage of 120 kVp in single-energy mode and varying tube currents, which were adjusted according to the volume CT dose index of a low-dose (2 mGy), standard-dose (6 mGy), and high-dose (12 mGy) scan protocols. The tube current was 25 mAs, 75 mAs, 149 mAs for PCD-CT and 35 mAs, 104 mAs, and 207 mAs for EID-CT, respectively. All scans were acquired with a pitch factor of 0.8, and a rotation time of 1000 ms.

### Reconstruction and post-processing

**Fig. 2:**
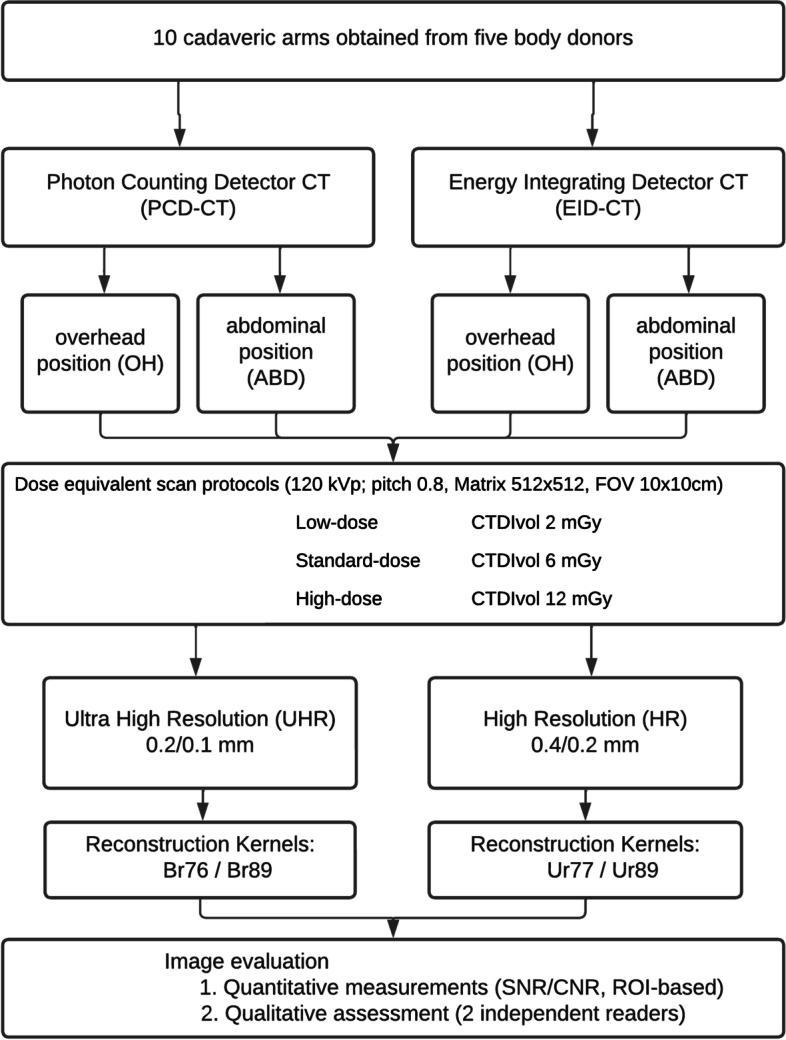
Flowchart of study setup and scanning parameters

All raw data from both systems were reformatted with a field-of-view (FOV) of 100 mm and image matrix of 512 × 512 aiming to achieve an identical in-plane resolution with a resulting maximum spatial frequency of 25.6 linepairs/cm [14]. The UHR scan modes were chosen: for the PCD-CT with a slice-thickness of 0.2 mm and increment of 0.1 mm, for the EID-CT with a slice thickness of 0.4 mm and increment of 0.2 mm. The reconstruction kernels (Br76 and Ur77) were chosen with bone analysis being the primary focus. In addition, the images were reconstructed with Br89 and Ur89 kernels, respectively, to compare the imaging performance using dedicated, very-sharp kernels beyond Ur77. The study setup is shown in a flow chart (Fig. 2). Detailed protocol parameters are summarized in Table 1.

**Table 1:** Acquisition and reconstruction parameters

	Photon-counting detector CT	Energy-integrating detector CT
Acquisition parameters		
Scanner^a^	NAEOTOM Alpha	SOMATOM Force
Collimation	24 × 0.2	19.2 × 0.6
Scan mode	UHR	UHR^b^
Tube potential (kVp)	120	120
Tube-current-time-product (mAs)	2 mGy (25 mAs) 6 mGy (75 mAs) 12 mGy (149 mAs)	2 mGy (35 mAs) 6 mGy (104 mAs) 12 mGy (207 mAs)
Rotation time (s/rotation)	1	1
Pitch factor	0.8	0.8
Reconstruction parameters		
Reconstruction type (image type)	QIR (T3D)	ADMIRE (UHR, 120 kV)
Iterative reconstruction strength (number of steps)	3	3
Slice thickness/increment (mm)	0.2/0.1	0.4/0.2
Field of view (mm)	100 × 100	100 × 100
Matrix size (no. of pixels)	512 × 512	512 × 512
Maximum image resolution^c^ (rho max; linepairs/cm)	25.6	25.6
Kernel parameters (rho10; linepairs/cm)	Br76 (21.01) Br89 (30.01)	Ur77 (21.99) Ur89 (30.01)

*ADMIRE* Advanced model-based iterative reconstruction, *Br* Body regular, *CT* Computed tomography, *QIR* Quantum iterative reconstruction, *T3D* Manufacturer’s name for the images created using photon energies from the low-energy threshold (20 keV) to the maximum possible energy (120 keV), *UHR* Ultra-high resolution, *Ur* Ultra-high-resolution regular (comb filter)

^a^All scanners manufactured by Siemens Healthineers GmbH, Forchheim, Germany

^b^With a highly attenuating, post-patient comb filter at the expense of radiation dose efficiency

^c^Maximum spatial frequency (rho max) that can be displayed in reconstructed images, determined by the field of view and the matrix size, approximated by the following equation: rho max = 1/(2 × FOV [cm]/matrix size [pixels]) based on the Nyquist theorem [14]

### Subjective image quality assessment

Two experienced radiologists with more than 5 years of experience in reading musculoskeletal images evaluated all CT images blinded and independently on a client-server based picture archiving and communication system workstation (Visage 7.1, Visage Imaging GmbH, Berlin, Germany). Image stacks covering the entire elbow reconstructed from both CT systems with different dose protocols and kernels respectively were randomly presented to the observers without technical details. The raters were allowed to use multiplanar reformations and 3D functionality of the review software. The initial window setting (contrast/width) was preset to 600/2,000 HU, however readers were allowed to adjust the contrast to their requirements. The readers were instructed to assess image quality on the basis of fracture and trabecular bone structures visualization. A 7-point rating scale was used for subjective evaluation of image quality as follows: 7 = excellent image quality, 6 = very good image quality, 5 = good image quality, 4 = satisfactory image quality, 3 = poor image quality, 2 = very poor image quality, and 1 = insufficient image quality/non-diagnostic image.

### Objective image quality assessment

**Fig. 3:**
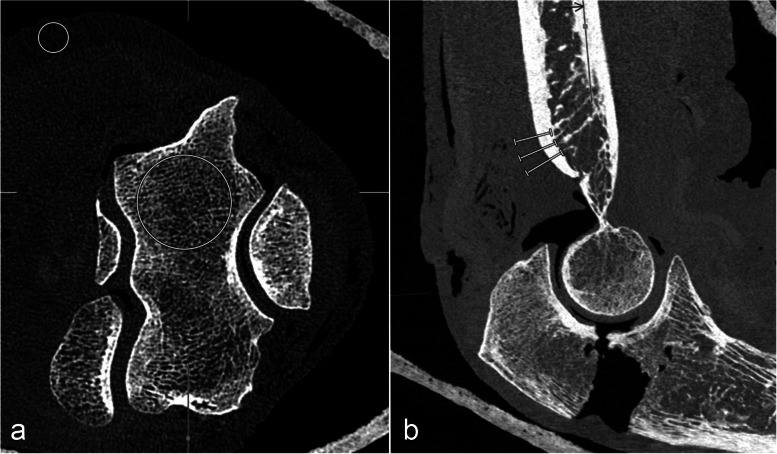
**a** Example of region of interest (ROI) placement for SNR/CNR measurements. The larger circle indicates the HU-measurements in the distal humerus. The smaller circle was placed in a homogeneous region of subcutaneous fat tissue and served as background for the calculations. Identical measurements were performed on three consecutive axial slices. **b** Example of cortical sharpness measurements with line profile analysis performed at the dorsal cortex of the humerus. *CNR* Contrast-to-noise ratio, *SNR* Signal-to-noise ratio

Identical regions of interest (ROIs) were placed within three consecutive axial CT slices in consistent locations of the humeral trabecular bone and in the adjacent subcutaneous fat tissue (Fig. 3a). Mean signal attenuation and standard deviation were recorded in Hounsfield units (HU) for each ROI. Noise was defined as the standard deviation of CT numbers within bone tissue. Signal-to-noise ratios (SNR) and contrast-to-noise ratios (CNR) were calculated as follows:

$$\textrm{SNR}={\textrm{HU}}_{\textrm{bone}}/{\textrm{SD}}_{\textrm{bone}}\ \textrm{and}\ \textrm{CNR}=\left({\textrm{HU}}_{\textrm{bone}}-{\textrm{HU}}_{\textrm{subcutaneous}\ \textrm{fat}}\right)/{\textrm{SD}}_{\textrm{bone}}.$$

Furthermore, objective measurements of cortical sharpness [15, 16] were computed for the 12-mGy scans in overhead arm position. The “line profile” function of the Visage review software was used to generate a HU-profile along a line perpendicular to the cortical bone of the dorsal humerus in sagittal reformations (three lines per cadaver as illustrated in Fig. 3b). The maximum HU differences were calculated for each tenth of a millimeter, the HU difference value per millimeter served as a parameter for cortical sharpness.

### Statistical analysis

All statistical analyses were calculated with SPSS Statistics 27.0.0.0 (IBM Corp., Armonk, NY, USA) Since there was no normal distribution of the continuous variables according to the Kolmogorov-Smirnov test, a Mann-Whitney *U* test was used for comparison of the SNR and CNR measurements. Reliability between raters was calculated using Cohen κ correlations for all measurements. The κ results were interpreted as follows: values ≤ 0 indicating no agreement, 0.01−0.20 as none to slight, 0.21−0.40 as fair, 0.41−0.60 as moderate, 0.61−0.80 as substantial, and 0.81−1.00 as almost perfect agreement [17]. Categorical variables were compared by the Wilcoxon signed ranked test. A *p* value lower than 0.05 was considered statistically significant.

## Results

**Fig. 4:**
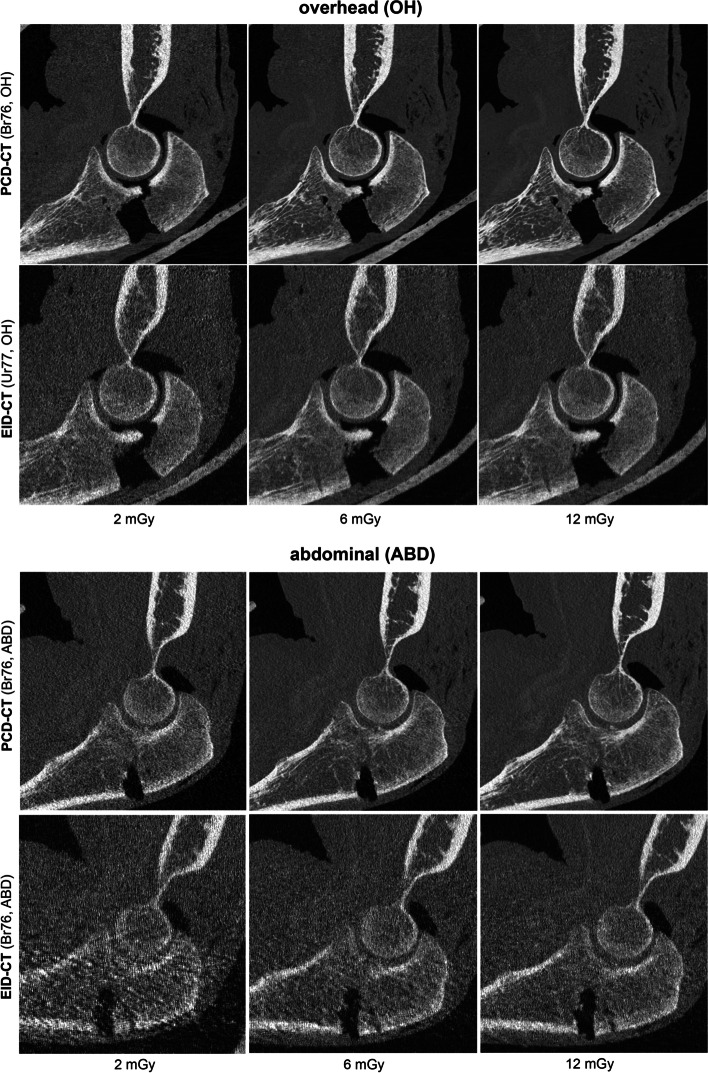
Comparison of photon counting detector (PCD)-CT and energy integrating detector (EID)-CT scans in overhead (OH) and abdominal (ABD) arm positions with Br76/Ur77 reconstruction kernels for low dose (2 mGy), standard dose (6 mGy), and high dose (12 mGy) scans

Two different arm scanning positions using three different dose protocols, each with the PCD-CT and the EID-CT resulted in a total of 120 elbow examinations in this study. Representative images are presented in Fig. 4.

### Subjective image quality assessment

A Cohen κ correlation coefficient of 0.672 with 0.24 standard error of the mean (*p* < 0.001) was obtained.

**Fig. 5:**
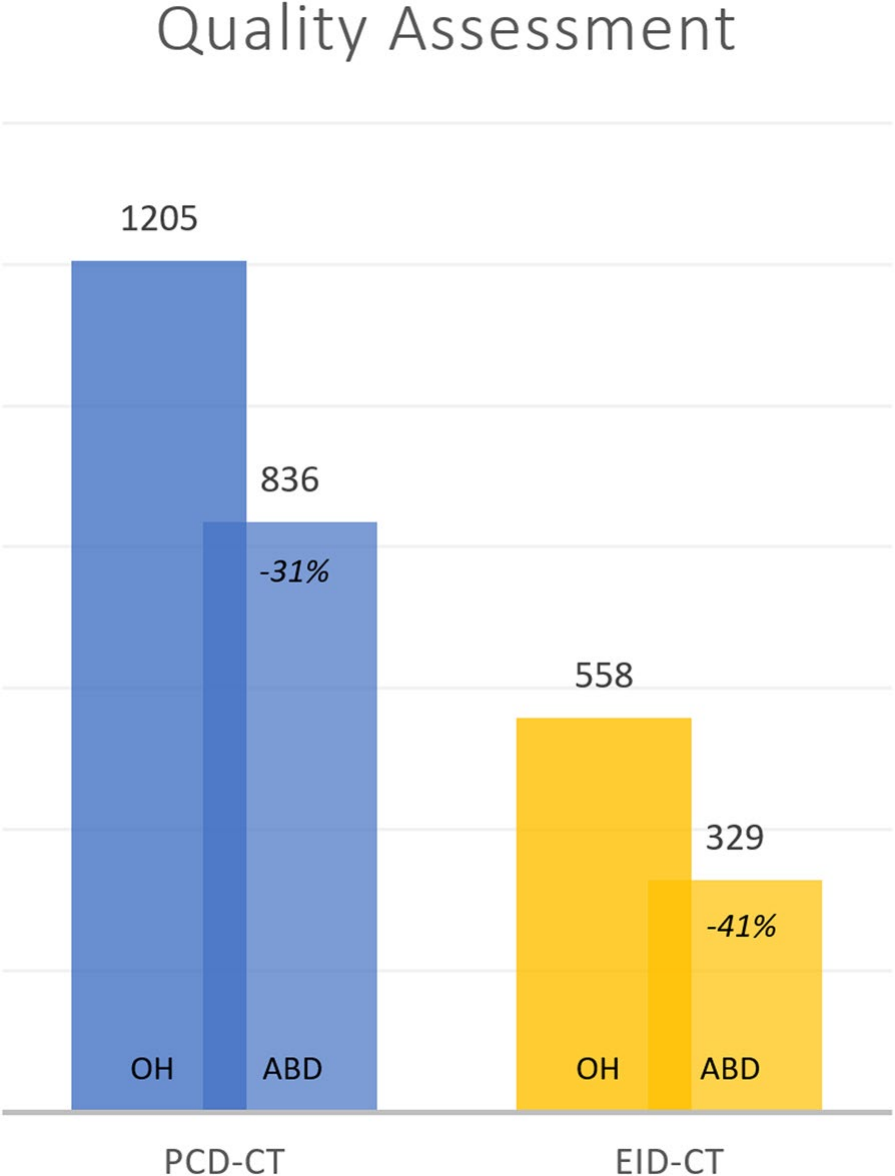
Cumulated absolute values of 7-point rating scale quality assessment, separately shown for different arm positions (*OH*, Overhead; *ABD*, Abdominal) and CT-scanners (PCD-CT blue, EID-CT yellow). The decrease of quality loss in abdominal arm positioning is proportionally lower (-31%) in PCD-CT compared to EID-CT (-41%). *EID-CT* Energy-integrating detector computed tomography, *PCD-CT* Photon-counting detector computed tomography

Almost 40% of PCD-CT rating values were very good (score 6) and excellent (score 7), while only 11% were nondiagnostic (score 1). In EID-CT about 62% ratings were nondiagnostic (score 1), the highest value was a good image quality (score 5) in about 2% of cases (Table 2). Cumulative values were significantly higher for PCD-CT compared to EID-CT, the decrease of image quality for an abdominal arm position compared to an overhead scan was proportionally lower (-31%) in PCD-CT than in EID-CT (-41%) (Fig. 5).

**Table 2:** Summarized quality assessment

Rating value	Photon-counting detector CT	Energy-integrating detector CT
1	54 (11.3%)	295 (61.5%)
2	86 (17.9%)	52 (10.8%)
3	50 (10.4%)	55 (11.5%)
4	64 (13.3%)	67 (14%)
5	39 (8.1%)	11 (2.3%)
6	95 (19.8%)	0 (0%)
7	92 (19.2%)	0 (0%)

Absolute and relative frequencies of quality assessment on a 7-point rating scale. Cumulative values for all scan parameters, cadavers, and readers are presented for the two computed tomography (CT) systems

**Fig. 6:**
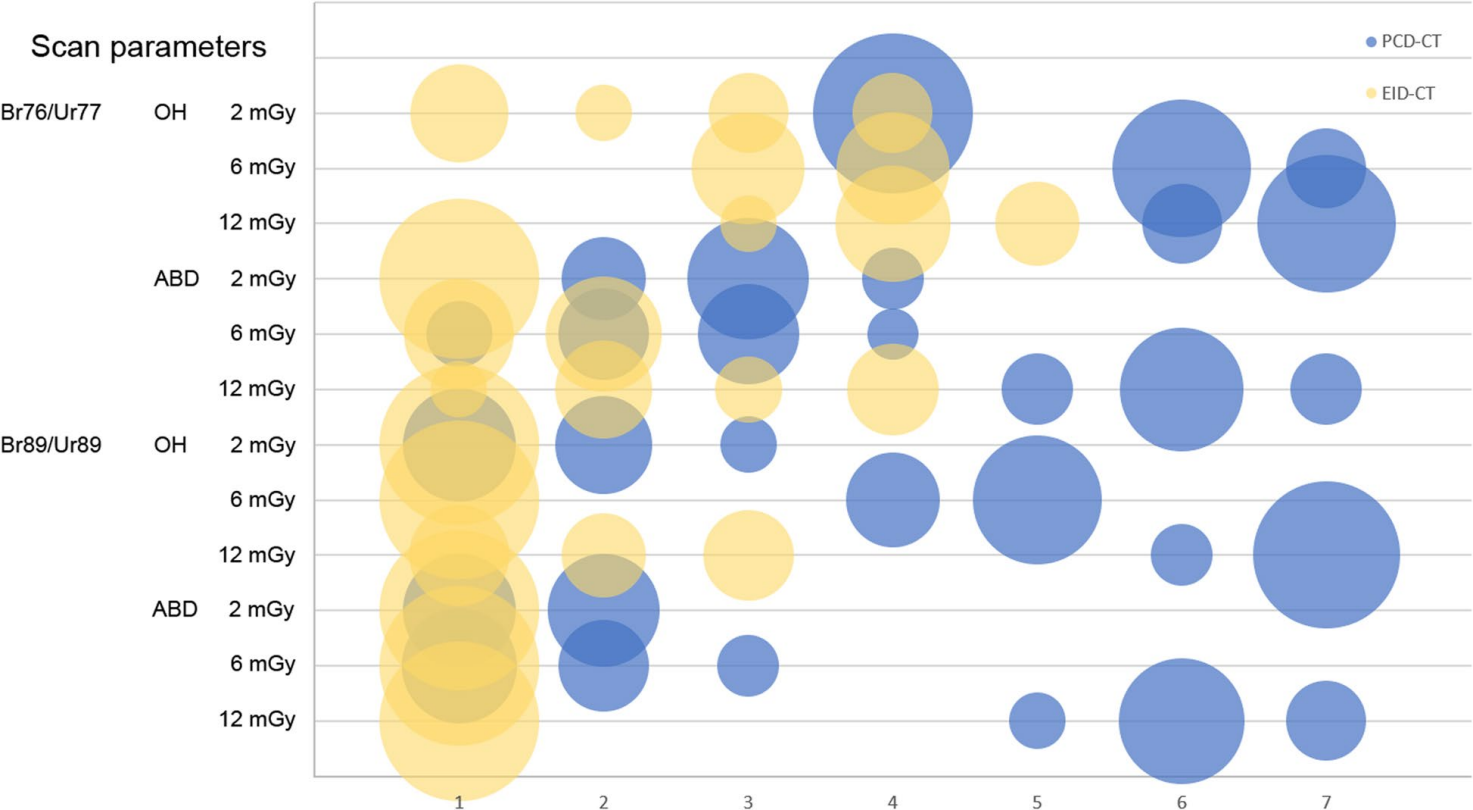
Quality assessment on a 7-point rating scale (*x* axis), cumulated for 10 arms, fracture, and trabecular bone assessment of two independent readers (*n* = 40) according to different scan parameters and arm positions (*y* axis). The area of each bubble indicates the frequency of the rating scale value chosen by the readers. The PCD-CT was evaluated better (blue) compared to the EID-CT (yellow). *EID-CT* Energy-integrating detector computed tomography, *PCD-CT* photon-counting detector computed tomography

Detailed results according to different scan settings (Table 3) show significantly better ratings for each protocol and arm position (*p* < 0.001), except for trabecular assessment with the Br89/Ur89 kernels at 2 mGy, which were considered nondiagnostic for both scanners. With EID-CT the Ur89 kernel was rated poor and very poor (rating scale 2−3) for fracture and trabecular bone assessment with high dose (12 mGy) and an overhead arm position. All other settings were nondiagnostic with the Ur89 kernel for EID-CT. The Br89 kernel with the PCD-CT provided the best results for fracture and trabecular bone evaluation at high scan dose (12 mGy). The distribution of the rating scale values according to scan settings and arm position is visualized for PCD-CT and EID-CT in Fig. 6.

**Table 3:** Detailed quality assessment

			Fracture assessment rating value (mean [min−max])			Trabecular bone assessment rating value (mean [min−max]		
Kernel	Position	Dose protocol	PCD-CT	EID-CT	*p* value	PCD-CT	EID-CT	*p* value
Br76/Ur77	OH	2 mGy	4.0 [4−4]	3.5 [3−4]	0.002	4.0 [4−4]	1.3 [1−2]^a^	< 0.001
		6 mGy	6.5 [6−7]	3.8 [3−4]	< 0.001	6.0 [6−6]	3.3 [3−4]	< 0.001
		12 mGy	7.0 [7−7]	4.6 [4−5]	< 0.001	6.5 [6−7]	3.8 [3−4]	< 0.001
	ABD	2 mGy	2.5 [2−3]	1.0 [1−1]^b^	< 0.001	2.5 [2−3]	1.0 [1−1]^b^	< 0.001
		6 mGy	3.2 [3−4]	1.9 [1−2]^a^	< 0.001	3.2 [3−4]	1.2 [1−2]^a^	< 0.001
		12 mGy	6.4 [6−7]	3.7 [3−4]	< 0.001	5.6 [5−6]	1.8 [1−2]^a^	< 0.001
Br89/Ur89	OH	2 mGy	2.3 [2−3]	1.0 [1−1]^b^	< 0.001	1.0 [1−1]^b^	1.0 [1−1]^b^	1.0
		6 mGy	4.3 [4−5]	1.0 [1−1]^b^	< 0.001	5.0 [5−5]	1.0 [1−1]^b^	< 0.001
		12 mGy	7.0 [7−7]	2.7 [2−3]	< 0.001	6.7 [6−7]	1.2 [1−2]^a^	< 0.001
	ABD	2 mGy	2.0 [2−2]	1.0 [1−1]^b^	< 0.001	1.0 [1−1]^b^	1.0 [1−1]^b^	1.0
		6 mGy	2.3 [2−3]	1.0 [1−1]^b^	< 0.001	1.7 [1−2]^a^	1.0 [1−1]^b^	< 0.001
		12 mGy	6.5 [6−7]	1.0 [1−1]^b^	< 0.001	5.8 [5−6]	1.0 [1−1]^b^	< 0.001

Quality assessment by two readers for 10 cadaveric specimens (mean value, min−max) for fracture and trabecular bone on a 7-point rating scale. The subjective evaluation was better for PCD-CT compared to EID-CT for almost all scan protocols. The *p* value of the Wilcoxon signed rank test analysis is reported

^a^Rating scale value: 1 (nondiagnostic), 2 (very poor)

^b^Rating scale value: 1 (nondiagnostic). *ABD* Abdominal scanning position, *EID-CT* Energy-integrating detector computed tomography, *OH* Overhead scanning position, *PCD-CT* Photon-counting detector computed tomography

### Objective image quality assessment

**Fig. 7:**
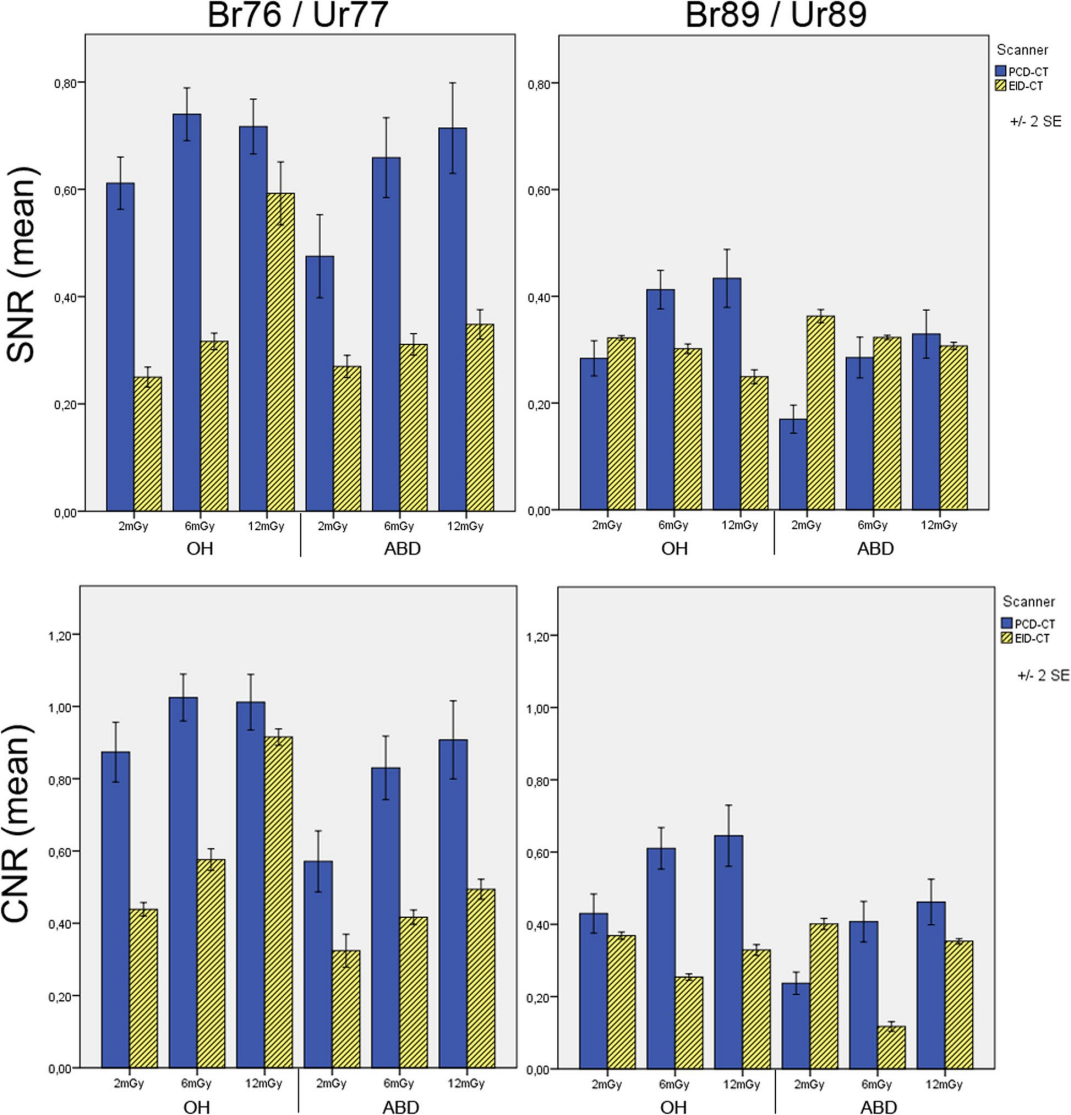
SNR (signal-to-noise ratio) and CNR (contrast-to-noise ratio) values for different reconstruction kernels, dose protocols, and scanning positions. *OH* Overhead, *ABD* Abdominal

For the Br76/Ur77 reconstruction kernels SNR and CNR were significantly higher for both arm positions for PCD-CT compared with the dose equivalent EID-CT (*p* < 0.001). For the Br89/Ur89 kernels significantly higher values for PCD-CT were measured for the 6-mGy and 12-mGy scan protocols with an overhead arm position for SNR and with overhead and abdominal positions for CNR. The other scan protocols showed no differences or higher SNR/CNR values for EID-CT compared to PCD-CT. Detailed results are presented in Table 4 and Fig. 7. Cortical sharpness at 12 mGy and the Br76/Ur77 kernels was measured significantly higher for the PCD-CT (1576 ± 199 ∆HU/mm, mean ± standard deviation) compared to EID-CT (1071 ± 152 ∆HU/mm), (*p* < 0.001). A further increase in image sharpness was possible with PCD-CT and the Br89 reconstruction kernel (1766 ± 369 ∆HU/mm). Reliable sharpness measurements were impossible for the Ur89 images of the EID-CT.

**Table 4:** SNR and CNR calculations

	Kernel	Position	Dose protocol	PCD-CT (mean ± SEM)	EID-CT (mean ± SEM)	*p* value
SNR	Br76/Ur77	OH	2 mGy	0.6 ± 0.11	0.3 ± 0.05	< 0.001
			6 mGy	0.7 ± 0.11	0.3 ± 0.04	< 0.001
			12 mGy	0.7 ± 0.11	0.6 ± 0.15	0.001
		ABD	2 mGy	0.5 ± 0.16	0.3 ± 0.05	< 0.001
			6 mGy	0.7 ± 0.16	0.3 ± 0.05	< 0.001
			12 mGy	0.7 ± 0.17	0.4 ± 0.07	< 0.001
	Br89/Ur89	OH	2 mGy	0.3 ± 0.07	0.3 ± 0.01	0.031
			6 mGy	0.4 ± 0.09	0.3 ± 0.02	< 0.001
			12 mGy	0.4 ± 0.12	0.2 ± 0.03	< 0.001
		ABD	2 mGy	0.3 ± 0.58	0.4 ± 0.03	< 0.001
			6 mGy	0.3 ± 0.08	0.3 ± 0.01	0.235
			12 mGy	0.3 ± 0.11	0.3 ± 0.02	0.774
CNR	Br76/Ur77	OH	2 mGy	0.9 ± 0.16	0.4 ± 0.04	< 0.001
			6 mGy	1.0 ± 0.14	0.6 ± 0.05	< 0.001
			12 mGy	1.0 ± 0.15	1.0 ± 0.12	0.013
		ABD	2 mGy	0.5 ± 0.32	0.4 ± 0.10	< 0.001
			6 mGy	0.9 ± 0.20	0.4 ± 0.04	< 0.001
			12 mGy	0.9 ± 0.21	0.5 ± 0.06	< 0.001
	Br89/Ur89	OH	2 mGy	0.4 ± 0.11	0.4 ± 0.03	0.031
			6 mGy	0.6 ± 0.13	0.3 ± 0.02	< 0.001
			12 mGy	0.7 ± 0.18	0.3 ± 0.04	< 0.001
		ABD	2 mGy	0.4 ± 0.71	0.4 ± 0.04	< 0.001
			6 mGy	0.4 ± 0.11	0.1 ± 0.03	< 0.001
			12 mGy	0.5 ± 0.13	0.4 ± 0.02	0.011

Calculations of SNR and CNR after a ROI-based measurement of signal attenuation for different dose protocols, arm scanning positions and reconstruction kernels. The results are given as mean values with the standard error of mean (**±** SEM). The *p* value of the Mann-Whitney *U* test analysis is reported. *ABD* Abdominal scanning position, *CNR* Contrast-to-noise ratio, *EID-CT* Energy-integrating detector computed tomography, *OH* Overhead scanning position, *PCD-CT* Photon-counting detector computed tomography, *ROI* Region of interest, *SNR* Signal-to-noise ratio

## Discussion

In this study, we compared the visualization of cadaveric elbow joints with fractures in a simulated post-trauma setting with off-center scanning positions between a PCD-CT and an EID-CT system. In dose equivalent scans, PCD-CT series were rated significantly better by two independent radiologists with regard to fracture and trabecular bone evaluation. SNR and CNR were measured significantly higher for all diagnostic PCD-CT images. Cortical sharpness was measured significantly higher for the PCD-CT compared to EID-CT for the Br76/Ur77 kernels. The arm scanning position, overhead *versus* abdominal, had less effect on image quality in the PCD-CT compared to the EID-CT. The application of a sharp bone kernel (Br89) resulted in improved image quality ratings in the PCD-CT series, whereas image quality with Ur89 in EID-CT was rated non-diagnostic or very low. In the low-dose scan mode, PCD-CT produced more diagnostic scans (75%) compared to EID-CT (19%).

The superiority of the PCD-CT becomes evident in the smaller number (11%) of nondiagnostic images compared to EID-CT (62%), suggesting a better compensation of difficult post-trauma scan situations, with substantial interrater reliability throughout the study. The major challenge are the lateral and sometimes vertical off-center positions of the elbow next to the body and joint flexion with the forearm oriented along the beam path. In particular, off-center positions significantly decrease image quality, increase image noise and radiation dose [18, 19]. A recent study aiming at protocol optimization for musculoskeletal UHR-CT reported a considerable effect of lateral phantom displacement on spatial resolution. The objective parameter for spatial resolution measurement, task transfer function 10% value [20], decreased 30.8% at maximal lateral displacement of only 8.5 cm [2]. Another study analyzed the effect of vertical off-centering and reported the highest image-noise values in patients positioned above the gantry isocenter [21]. In the last years, progress has been made in the field of (semi-)automatic patient positioning, *e.g.,* based on patient surface detection by means of 3-dimensional cameras or possibilities of lateral table displacement [22–24]. Still, the algorithms are mostly applicable rather to central than to peripheral body parts. Individualized approaches, focusing on musculoskeletal applications, remain challenging.

Novel gantry-free cone-beam CT options are worth mentioning as an alternative for elbow imaging. A recent study demonstrated an improved diagnostic performance for the diagnosis of fractures and fracture-related findings in elbow joint examinations compared to two-dimensional radiography [25]. Probably, crucial factors limiting image quality compared to gantry CT systems could be the individual size of the patients’ extremities and the amount of beam hardening artifacts.

In gantry CT systems bowtie filter geometry and angular sampling principles are optimized for evaluating round or elliptical objects at the center of the gantry [5]. Hence, due to the off-center positioning, the elbow joints in our study were considerably exposed to a reduced x-ray intensity towards the edges of the gantry. According to our results, the PCD-CT system is capable to compensate these limitations and to produce a better image quality when compared to EID-CT. This is most likely related to the detector design which does not require the generation of visible light, but creates a detector signal from the energy of each photon. Hence, especially lower-energy x-rays are not down-graded during the averaging process inside each detector pixel. On the contrary, each x-ray is measured individually resulting in an equal contribution of low and high energy x-rays [6]. The advantages of the PCD-CT are most obvious at lower scan doses. Another major benefit of the innovative detector design is the threshold-based elimination of baseline electronic noise at 20 keV which is especially beneficial for low-dose imaging and obese patients [6].

Moreover, the detector design of the PCD allows scanning in ultra-high resolution modes with small pixel sizes without signal loss due to optical separation or the application of comb filters. Earlier studies were able to show that a noise reduction of 30–40% can be achieved when optical separation is not required [26, 27]. A study with cadaveric wrists demonstrated substantially higher SNR and CNR in PCD-CT compared to EID-CT [28]. Furthermore, improved image quality at similar doses or the potential of dose reduction of PCD-CT compared to EID-CT systems has been described for abdominal and chest CT scans [9, 29–32]. Our study supports these results for imaging of the elbow joint and emphasizes the importance of accurate patient positioning. The image quality decrease that was observed with EID-CT when changing the arm scanning position from overhead to abdominal was less pronounced with PCD-CT, suggesting a better compensation of disadvantageous scanning positions.

The PCD-CT images reconstructed with a dedicated sharp kernel and smaller slice thickness demonstrated better fracture and trabecular bone delineation. The best image quality was achieved with the very-sharp kernel (Br89) in PCD-CT, whereas the very-sharp kernel (Ur89) in EID-CT significantly increased image noise and mostly resulted in non-diagnostic images. As indicated by the calculated rho values and kernel parameters (Table 1), an additional increase in image resolution/sharpness would have been possible in PCD-CT with a 1024 × 1024 matrix. However, we chose a 512 × 512 matrix size for both systems for reasons of comparability and noise reduction. The use of very-sharp kernels is an obvious advantage of PCD-CT for musculoskeletal applications, mostly due to the better intrinsic system resolution with smaller pixels and the absence of inter-pixel septa or a comb filter. Alternatively, the increased geometric dose efficiency can be used to reduce the radiation dose compared to EID-CT when image quality or noise are matched. A recent study reported superior objective and subjective image quality characteristics of PCD-CT over EID-CT for the delineation of tiny bone details in an animal model with mice [33]. A clinical study could show that UHR imaging with PCD-CT depicts wrist structures more clearly than conventional EID-CT despite a 49% dose reduction [34]. Another clinical study demonstrated an improved visualization of osseous structures of shoulders and pelvises in UHR PCD-CT at a 31–47% lower radiation dose compared to EID-CT [35]. However, experience with musculoskeletal applications is still limited and more studies are necessary to elucidate the best scanning parameters for each joint in clinical routine. In the future, we assume further improvements of PCD-CT images owed to the continuous software development.

A limitation of our study is the preclinical design with cadaveric specimens and a standardized reference phantom. Patient size differences and respiratory or motion artifacts could not be examined, the actual relevance for clinical applications is limited. Bone and soft tissue change due to fixation could have influenced proper evaluation. Fracture assessment could be altered compared to *in vivo* as the fracture gap mostly included air. In addition, the olecranon fracture was very evident, the diagnostic accuracy of subtle fractures was not evaluated. Another important limitation is the comparability of different iterative reconstruction algorithms (QIR *versus* ADMIRE). In addition, kernels were not identical but matched as close as possible between both CT systems according to the rho 10 values. There were limitations in the quantitative measurements for the very sharp reconstructions of the EID-CT which needs to be considered for result interpretation. We assumed that the ROI-based SNR or CNR measurements as well as the line profile analysis for the Ur89 EID-CT images are not reliable and were adversely affected by the very low image quality and high image noise. Finally, CT scanners of only one manufacturer were investigated for accessibility reasons.

In conclusion, ultra-high resolution PCD-CT showed superior objective and subjective image quality characteristics over EID-CT for delineation of trabecular bone and olecranon fractures with an off-center arm position in a simulated post-trauma setting. The major advantage of PCD-CT for musculoskeletal imaging is the use of very-sharp reconstruction kernels leading to high image quality, even under difficult scanning conditions.

## Data Availability

The datasets used and/or analyzed during the current study are available from the corresponding author on reasonable request.

## Abbreviations

3D: Three-dimensional
CT: Computed tomography
EID-CT: Energy-integrating detector CT
FOV: Field of view
HU: Hounsfield units
PCD-CT: Photon-counting detector CT
ROI: Region of interest
UHR: Ultra-high resolution
